# Stability of Feline Coronavirus in aerosols and dried in organic matrices on surfaces at various environmental conditions

**DOI:** 10.1038/s41598-023-49361-1

**Published:** 2023-12-12

**Authors:** Janina Reissner, Paul Siller, Alexander Bartel, Uwe Roesler, Anika Friese

**Affiliations:** 1 Institute of Animal Hygiene and Environmental Health, Veterinary Centre for Resistance Research—TZR, School of Veterinary Medicine, Freie Universität Berlin, 14163 Berlin, Germany; 2 Institute of Veterinary Epidemiology and Biostatistics, School of Veterinary Medicine, Freie Universität Berlin, 14163 Berlin, Germany; 3 Present Address: Federal Office of Consumer Protection and Food Safety, Department Veterinary Drugs, Mittelstraße 51-54, 10117 Berlin, Germany

**Keywords:** Viral transmission, SARS virus, Virology, SARS-CoV-2, Air microbiology, Pathogens

## Abstract

Enveloped respiratory viruses, including the severe acute respiratory syndrome coronavirus 2 (SARS-CoV-2), can be transmitted through aerosols and contact with contaminated surfaces. The stability of these viruses outside the host significantly impacts their transmission dynamics and the spread of diseases. In this study, we investigated the tenacity of Feline Coronavirus (FCoV) in aerosols and on surfaces under varying environmental conditions. We found that airborne FCoV showed different stability depending on relative humidity (RH), with higher stability observed at low and high RH. Medium RH conditions (50–60%) were associated with increased loss of infectivity. Furthermore, FCoV remained infectious in the airborne state over 7 h. On stainless-steel surfaces, FCoV remained infectious for several months, with stability influenced by organic material and temperature. The presence of yeast extract and a temperature of 4 °C resulted in the longest maintenance of infectivity, with a 5 log_10_ reduction of the initial concentration after 167 days. At 20 °C, this reduction was achieved after 19 days. These findings highlight the potential risk of aerosol and contact transmission of respiratory viruses, especially in enclosed environments, over extended periods. Studying surrogate viruses like FCoV provides important insights into the behavior of zoonotic viruses like SARS-CoV-2 in the environment.

## Introduction

The importance of understanding the tenacity of respiratory viruses, particularly coronaviruses, in the environment has been highlighted by the recent severe acute respiratory syndrome coronavirus 2 (SARS-CoV-2) pandemic. The stability of these viruses in the environment has a direct influence on their transmission and spread of diseases. Coronaviruses (CoVs) are enveloped positive single-stranded RNA viruses^1^. They have major medical, veterinary, and economic relevance, as they can infect various mammals and birds, depending on the genus. In these hosts CoVs can cause a wide spectrum of mild to serious diseases, often associated with high morbidity and mortality^2^. In our study, we used Feline Coronavirus (FCoV) as a surrogate virus for SARS-CoV-2. Another research group recently emphasized the importance of studying non-zoonotic animal CoVs to understand the behavior of zoonotic CoVs^3^. Respiratory viruses can be transmitted through three different routes: droplet, aerosol (droplet nuclei), and contact transmission^4–6^. At the beginning of the pandemic, the primary transmission routes of SARS-CoV-2 as a respiratory virus were a subject of frequent discussion^7^. While the focus was initially on droplet transmission, aerosol transmission was later confirmed as the predominant transmission route^8,9^. For a pathogen to be transmitted through these routes, it has to remain airborne and infectious outside the host for a certain duration. Respiratory activities such as breathing, talking, singing, coughing and sneezing generate particles of various sizes and quantities, which may contain the pathogen^10^. Larger respiratory droplets carrying infectious agents, predominantly expelled by symptomatic infected individuals through coughing or sneezing, sediment within 1–2 m of the source in seconds^11,12^. These droplets either directly reach the mucous membranes or conjunctiva of susceptible hosts or sediment on nearby surfaces^13^. If the virus remains infectious on these surfaces after drying for a certain duration, individuals may become infected through contact with the contaminated surface. Previous studies found that SARS-CoV-2 remained infectious for 4–7 days on stainless-steel surfaces at room temperature^14,15^. Additionally, another study reported a prolonged infectious period of SARS-CoV-2 on stainless-steel surfaces of 14 days when the virus was embedded in an organic matrix^16^. It is also likely for larger droplets to evaporate into droplet nuclei before reaching the ground. The particle diameter that distinguishes larger droplets from droplet nuclei is usually considered to be 5 µm, although some studies have used a cut-off size of 10 µm as these particles are able to deposit in the lower respiratory tract^6,17^. Previous studies have demonstrated that droplet nuclei can remain suspended in the air for hours or even days, travel longer distances from the source, and accumulate in enclosed environments^11,12^. SARS-CoV-2 has been found to remain infectious in aerosol particles for several hours^14,18^. Aerosol transmission occurs when these small infectious aerosol particles, produced by infected hosts during respiratory activities, whether symptomatic or asymptomatic, are inhaled^19^. Exhaled particles undergo evaporation at a rate, which depends on the particle surface, the ambient air temperature and relative humidity (RH)^12,17^. Evaporation changes the diameter, physical state and chemical composition of the particles, potentially influencing the stability of the enclosed virus, which may be harmed^20^. Therefore, virus stability is highly affected by environmental conditions, including ambient RH, temperature, as well as factors like UV radiation and oxidative stress. However, the exact mechanisms of viral inactivation are still not fully understood and are very specific to each virus^21^. The wide variability in physical and biological properties among viruses makes it challenging to generalize findings across different pathogens. Comprehensive analyses of numerous pathogens are necessary to understand the effects of environmental factors on their stability and to identify possible correlations. In this study, our focus was on determining the influence of RH on the stability of airborne FCoV. Additionally, we investigated time-dependent loss of infectivity of airborne FCoV in an enclosed environment under moderate climatic conditions. Furthermore, we examined the stability of FCoV, embedded and dried in different organic matrices, on stainless-steel surfaces under different environmental conditions.

## Materials and methods

### Virus propagation and quantification

An FCoV I strain, biotype FECV (isolate “München”), provided by the Friedrich Loeffler Institute (FLI, Insel Riems, Germany; viral registration number RVB-1259), was used for all experiments. FCoV was propagated and titrated using Crandell-Rees Feline Kidney Cells (CRFK; ATTC CCL-94) as described previously^22^. The cells were grown in Dulbecco’s Modified Eagle Medium (DMEM High Glucose, Biowest, Nuaillé, France) supplemented with 10% fetal bovine serum (FBS; PAN Biotech, Aidenbach, Germany) and 1% of a solution containing 100 IU/mL penicillin G, 100 µg/mL streptomycin (Biochrom AG, Berlin, Germany) and 25 µg/mL Amphotericin B (Biozym, Hessisch Oldendorf, Germany). Virus quantification was performed as described recently^22^. In brief: we performed endpoint dilution assays. The tissue culture infectious dose 50 (TCID_50_/mL) was calculated using the Spearman-Karber method^23,24^ after virus titration in 96-well plates (Sarstedt, Nürmbrecht, Germany) in an eightfold approach. Every well was investigated for a cytopathic effect five days after infection.

### Aerosol chamber and bioaerosol generation

The aerosol experiments were performed in an airtight walk-in aerosol chamber with a volume of 7 m^3^. Further technical details concerning the aerosol chamber were published previously^25^. In brief, the bioaerosol was generated by an ultrasonic nebulizer (Broadband Ultrasonic Generator, SonoTek Corporation, Milton, MA, United States), producing particles with an average initial size of 18 µm. A virus suspension containing FCoV in DMEM and 10% FBS was transported to the ultrasonic nebulizer using a perfusor pump at a flow rate of 36 mL/h. The concentration of the original FCoV suspension was calculated after each experiment and based on this the theoretically possible concentration of viruses per m^3^ air was calculated. The chamber can be operated in different modes, resulting in dynamic or static aerosols. In the dynamic aerosol, there was continuous air exchange at a rate of 100 m^3^/h, and a fan installed in the ceiling of the chamber distributed the aerosol with a rotation speed of 3770 rpm. On the other hand, in the static aerosol, there was no air exchange, and the fan was inactive. Climate parameters such as RH and temperature could be individually regulated in the dynamic aerosol, as they were set in the chamber supply air.

### Air sampling and quantification of the air samples

We used the Coriolis µ cyclone air sampler (Bertin Instruments, Montigny-le-Bretonneux, France) for air sampling. The total sample volume was 3000 L, using an air flow rate of 300 L/min over a sampling time of 10 min. Depending on the type of aerosol, the air samplers were either placed inside the chamber (static aerosol) or connected to the chamber from outside using a stainless-steel tube, enabling air collection from the center of the chamber (dynamic aerosol). In the case of static aerosol, the samplers were placed inside the chamber to avoid the generation of negative pressure that would result from air extraction without incoming airflow. We conducted preliminary tests to compare collection efficiencies, and both setups achieved similar results, ensuring accurate air sampling. The Coriolis µ cones were filled with 10 mL of supplemented DMEM. Unlike the cell culture medium, we added only 1% of the FBS. In addition, 0.3% autoclaved linseed oil was added to prevent foam formation. Following sampling, the air samples and the remaining original virus suspension, which had been aerosolized, were stored on ice until quantification using an endpoint dilution assay, as described previously.

### Influence of RH on the stability of airborne FCoV

In the first part of this study, we examined the stability of aerosolized FCoV under varying RH conditions. Specifically, we investigated the stability at 30%, 40%, 50%, 60%, and 70% RH in a fivefold approach. The temperature was adjusted to 24 °C throughout each trial. We generated a dynamic aerosol in the chamber with continuous air exchange at a rate of 100 m^3^/h, while introducing the virus aerosol into the chamber at a perfusor pump flow rate of 36 mL/h throughout the entire experiment. The ultrasonic nebulizer, air exchange and the fan were started 8 min before air sampling to establish a homogeneous viral aerosol in the chamber. They continued to run for the entire sampling period, in total 18 min, enabling a more precise assessment of the impact of RH levels and minimizing sedimentation losses. To confirm a good distribution of the aerosol in the chamber, we collected two simultaneous air samples using two Coriolis µ samplers connected to the chamber at different heights. The heights were 1.6 m and 0.8 m. All samples were quantified using an endpoint dilution assay, as described previously.

### Influence of time on the stability of airborne FCoV

In the second part of this study, we focused on investigating the natural loss of infectivity of aerosolized FCoV over time. Our aim was to produce a static aerosol to simulate an enclosed environment without any air exchange where a virus emitter was present and to investigate how the viral load in the air changes over time. The ultrasonic nebulizer operated for 10 min at a perfusor pump flow rate of 36 mL/h to generate a concentrated viral aerosol within the chamber. After the initial aerosolization, the virus supply was stopped, and the aerosol remained in the chamber for several hours without any further airflow. Air samples were collected after 10, 60, 100, 180, 300, and 420 min in a fivefold approach, respectively. A new experiment was conducted for each time point to prevent previous air sampling from affecting the results. We recorded the RH and temperature in the chamber using a Testo 400 probe (Testo Ltd, Alton Hampshire, UK). As there was no supply air, we were unable to regulate the climate parameters individually. The average RH in the chamber was 33%, and the temperature was 23 °C throughout all experiments. Furthermore, the particle concentration in the aerosol was measured by an aerosol spectrometer (Grimm, model 1.109, GRIMM Aerosol Technik Ainring GmbH & Co., KG, Germany). Additionally, as part of our investigation, we implemented sedimentation plates to collect any virus particles that settled over time. Therefore a 12 × 12 cm plate (Greiner Bio-One GmbH, Frickenhausen, Germany), filled with 20 mL of supplemented DMEM, was placed at the center of the chamber for each trial. All samples were quantified using an endpoint dilution assay, as described previously.

### Influence of different environmental conditions on the stability of FCoV dried in organic matrices on surfaces

In a further experimental series, we investigated the stability of FCoV when dried in organic matrices on stainless-steel germ carriers, subjected to different environmental conditions. The round stainless-steel germ carriers (GK Formblech, Berlin, Germany) were manufactured from 1.4301-2B (X5CrNi1810) steel, with a diameter of 2 cm and a thickness of 1 mm and have a flat surface. To load the germ carriers, we combined 100 µL of FCoV suspension with 11 µL of two different organic loads in a 10:1 ratio. The first organic load consisted of 100 g/L yeast extract (Merck, Darmstadt, Germany) and 100 g/L Bovine Serum Albumin (BSA; Sigma, St Louis, Missouri), resulting in a final concentration of 10 g/L per germ carrier. The second organic load consisted of 30 g/L defibrinated sheep blood (Thermo Fisher Scientific, Waltham, Massachusetts) and 30 g/L BSA, resulting in a final concentration of 3 g/L per germ carrier. Both organic load solutions were previously sterilized and mixed. Following a drying period of 45 min in a desiccator, the carriers were stored at either 20 °C or 4 °C until further use. To determine the initial virus concentration on day 1 (TCID_50/_germ carrier), triplicate germ carriers were investigated immediately after drying. Subsequently, triplicate germ carriers were examined at each designated time point. The dried virus suspension was resuspended in 1 mL of DMEM with the addition of 3% FBS and 0.3% autoclaved linseed oil by adding glass beads (Rettenberg, Goettingen, Germany) and vortexing for 1 min. An endpoint dilution assay was then performed, following previously described methods. For the yeast extract/BSA organic load, the time points were as follows: daily on days 1, 2, 3, and 4; every second day on days 6, 8, and 10; every third day on days 13, 16, 19, 22, and 25; and approximately weekly on days 31, 40, 50, and 60. After 60 days, a second series of trials was started because we were out of germ carriers as we underestimated the stability of FCoV. The new germ carriers were examined at approximately weekly to every second-week intervals on days 6, 25, 60, 85, 98, 105, 111, 125, 134, 140, 147, 162, 168, and 174, or until no infectious virus was detectable. For the sheep blood/BSA organic load, the time points were as follows: daily on days 1, 2, 3, and 4; every second day on days 6, 8, and 10; every third day on days 13, 16, 19, 22, and 25; and approximately weekly on days 31, 35, 40, 50, and 55, or until no infectious virus was detectable.

### Statistical analysis

All statistical analysis was performed using R version 4.2.2 (R Foundation Vienna). Since viral TCID_50_ were lognormal distributed, we report the log_10_ TCID_50_. Results are reported with 95% confidence intervals. A significance threshold of 0.05 was used.

To model the effect of the RH on the recovered FCoV TCID_50_/m^3^ air we used a mixed count regression with log link. Due to overdispersion we choose a negative binomial distribution. The TCID_50_/m^3^ was modelled depending on RH and adjusted for Coriolis height, both variables were included as categorical effects. Post-hoc comparisons between all RH were adjusted for multiple comparisons using the Bonferroni method. Estimated marginal means and multiple comparison post-hoc tests were performed using the emmeans R package (version 1.8.6). For visualization we modelled the TCID_50_/m^3^ depending in the relative humidity as a continuous effect using a restricted cubic spline with the actually measured RH with the package mgcv (version 1.8-42).

The influence of time on the airborne FCoV TCID_50_/m^3^ air was modelled as an exponential decay (loss of infectivity) using a Bayesian loglinear model.

$$log_{{10}} \left( {{\text{FCoVin}}\frac{{TCID_{{50}} }}{{{\text{m}}^{3} {\text{air}}}}} \right) = {\text{initial}}\;{\text{conc}} + {\text{time}}$$

The *initial conc* (intercept) is the estimated aerosolized log_10_ TCID_50_/m^3^ concentration of FCoV at 0 min and *time* is the reduction in log_10_ TCID_50_/m^3^ per min (i.e. exponential decay). For the stability of FCoV on surfaces we had to add a loglog linear effect to account for the longer survival of FCoV due to the organic matrix/load (yeast extract/BSA or sheep blood/BSA). One model was fitted for each temperature and organic load combination.

$$log_{{10}} \left( {{\text{FCoVin}}\frac{{TCID_{{50}} }}{{{\text{m}}^{3} {\text{air}}}}} \right) = {\text{initial}}\;{\text{conc}} + {\text{time}} + {\text{log}}({\text{time}})$$

The slowing rate of decay was modelled with *log(time).* This effect represents the longer survival of virus which is protected from environmental factors by being embedded in the organic matrix. Both models were fitted using brms (version 2.19.)^26^ and rstan (version 2.26.13). Time until log reduction was calculated based on these models.

## Results

### Influence of RH on the stability of airborne FCoV

**Figure 1:**
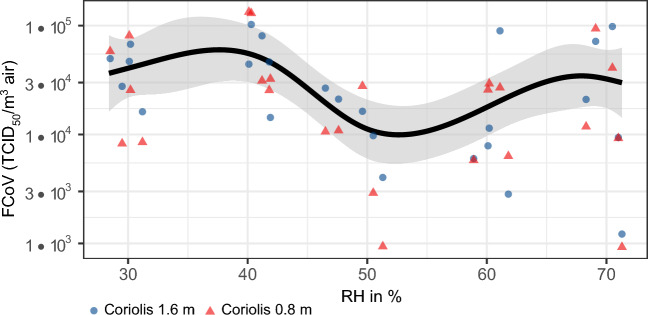
Stability of aerosolized FCoV at different RHs. The black line shows the predicted concentration of airborne FCoV *in TCID*_*50*_*/m*^*3*^ at different RH levels. The symbols represent the actual measurements of RH and FCoV concentration at 1.6 m Coriolis height (blue circles) and 0.8 m Coriolis height (red triangles). Each experiment was repeated five times. The shaded area indicates the upper and lower 95% confidence interval.

The stability of aerosolized FCoV as a function of RH (30–70%) in dynamic aerosols is shown in Fig. 1. The concentration of the original FCoV suspension was mean (M) = 6.26 (SD 0.47) log_10_ TCID_50_/ml for each trial. After a stabilization time of the aerosol of 8 min, air samples were simultaneously collected at two different heights (1.6 m and 0.8 m) in each trial. Overall, there was no significant difference in the amount of collected infectious viruses between the two heights (*p *= 0.789). FCoV was most robust at 40% RH, with a concentration of M = 4.72 (SD 0.30) log_10_ TCID_50_/m^3^ air. Conversely, FCoV was most unstable at 50% RH, with a concentration of M = 3.99 (SD 0.50) log_10_ TCID_50_/m^3^ air. Significant differences were observed between 40 and 50% RH (*p* = 0.009) as well as between 40 and 60% RH (*p* = 0.020). Additionally, we calculated recovery rates of FCoV in the aerosol by dividing the concentration of collected FCoV per m^3^ of air by the theoretical concentration of viruses per m^3^ of air, which was previously calculated from the original FCoV concentration in the suspension. The maximum average recovery rate of FCoV in the aerosol was 13.3% at 40% RH, while the minimum average recovery rate was 2.5% at 50% RH.

### Influence of time on the stability of airborne FCoV

**Figure 2:**
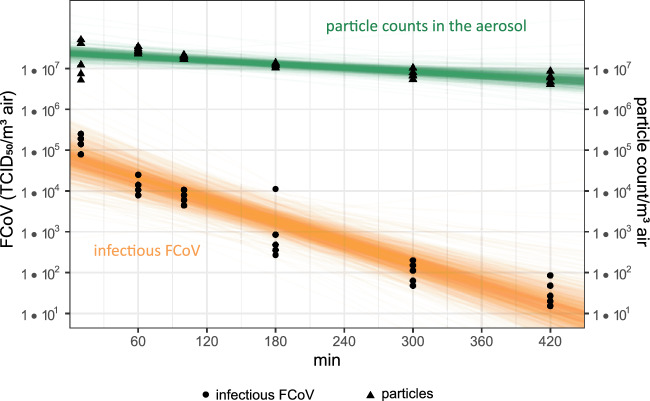
Loss of infectivity of aerosolized FCoV over time. The concentration of infectious FCoV (TCID_50_/m^3^) is shown on the left y-axis and the counted particles/m^3^ are shown on the right y-axis. Both are plotted on a logarithmic scale. Circles represent the actual measurements of the infectious FCoV concentration and triangles represent the raw measurements of the particle counts in the aerosol. The orange and green lines indicate posterior draws (uncertainty) of the predicted decay of infectious FCoV and particles from the Bayesian log linear regression model.

Furthermore, we investigated the stability of airborne FCoV in a static aerosol over time without any additional external influences. The loss of infectivity of FCoV over time and the corresponding total particle count is shown in Fig. 2*.* The average RH in the chamber was 33%, and the temperature remained constant at an average of 23 °C throughout all experiments. The concentration of the original FCoV suspension was M = 6.15 (SD 0.31) log_10_ TCID_50_/mL for each trial. After 10 minutes (min) of aerosolization (t0), we detected an initial FCoV concentration of M = 5.20 log_10_ TCID_50_/m^3^, resulting in a recovery rate of 13% at t0. Afterwards, the initial concentration decreased over time linearly with a rate of − 0.009 log_10_ TCID_50_/m^3^ per min (95% CI [− 0.012, − 0.005]) or − 2% per min, which corresponds to a half-life of 34.8 min (95% CI [24.7, 57.3]). After median (Md) = 117 min (95% CI [85, 195]), the initial concentration decreased by 1 log_10_ level. After Md = 349 min (95% CI [252, 584]), the initial FCoV concentration decreased by 3 log_10_ levels, corresponding to a recovery rate of only 0.01%. The experiment was terminated after 420 min, as we had almost reached the detection limit of the assay of 0.8 log_10_ TCID_50_/m^3^. According to the model, a 4 log_10_ reduction of the initial concentration was predicted after Md = 464 min (95% CI [336, 778]). At the same time, we conducted measurements of the aerosol particles throughout the entire experiment to assess any potential loss of particles, including viruses, from the air. The aerosol spectrometer detected the highest concentration of total particles at M = 7.8 log_10_ particles/m^3^ after 15 min. The particle count remained relatively stable, with only a 1 log_10_ level decrease over the 420 min duration. Additionally, we used sedimentation plates to collect infectious virus particles that sedimented over time. Table 1 presents the concentration of sedimented infectious FCoV in log_10_ TCID_50_/mL at various time points. The concentration averaged M = 3.02 (SD 0.09) log_10_ TCID_50_/mL and remained relatively constant at every time point. To estimate the proportion of infectious virus particles that sedimented on the base area of the chamber, we extrapolated the concentration per ml to the entire chamber base area and divided it by the theoretical concentration of viruses in the chamber calculated from the original FCoV suspension. We found that M = 31.4% of infectious virus particles immediately sedimented and were no longer available for the air sampler. Notably, this value remained consistent across all time points.

**Table 1:** Concentration of sedimented infectious FCoV at different time points.

Time points (min)	FCoV concentration (log_10_ TCID_50_/mL)
10	2.96
60	2.96
100	3.14
180	3.00
300	2.92
420	3.11

### Influence of different environmental conditions on the stability of FCoV dried in organic matrices on surfaces

**Figure 3:**
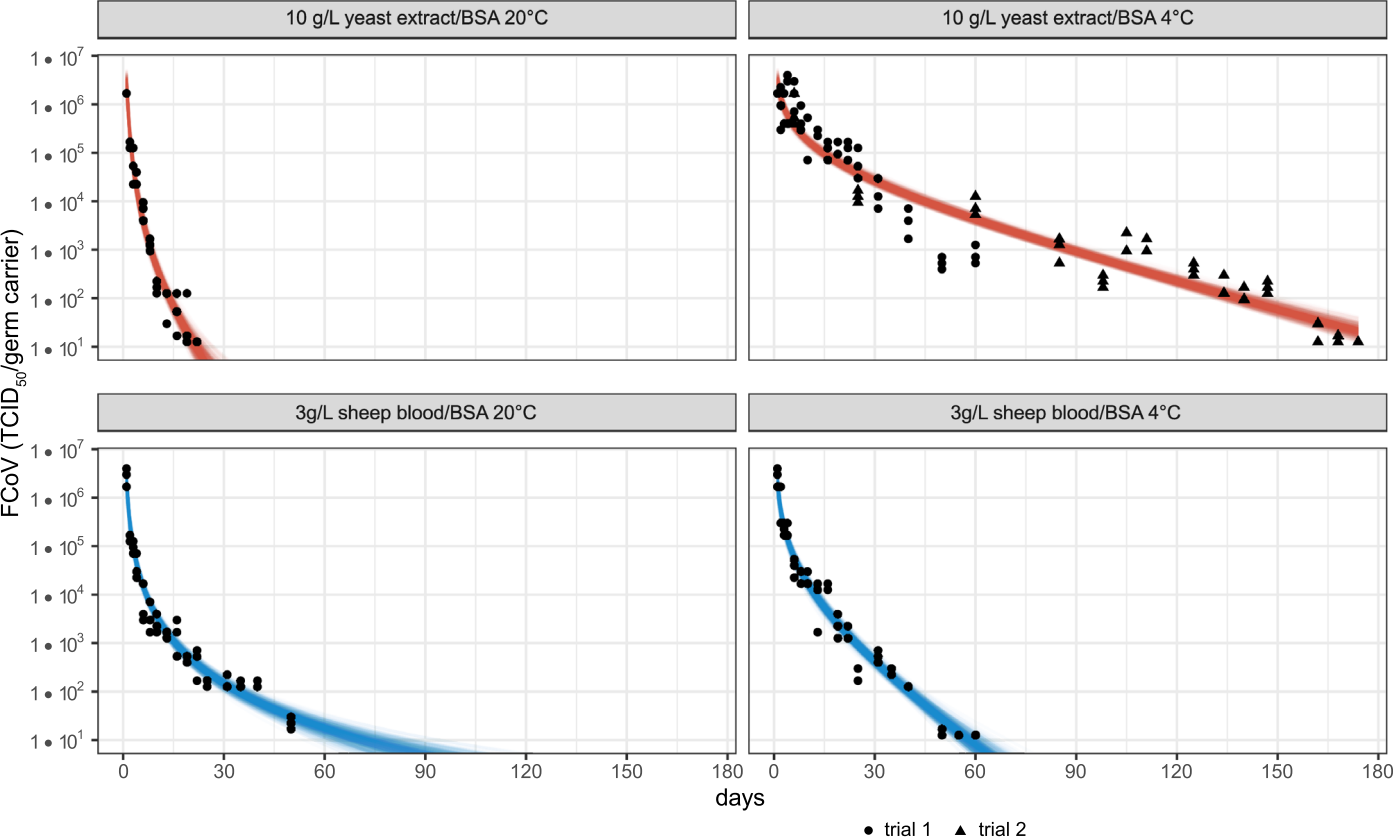
Loss of infectivity of FCoV on stainless-steel germ carriers over time. The upper graphs show the loss of infectivity of FCoV with a 10 g/L yeast/BSA organic load at 20 °C (left) and 4 °C (right). The lower graphs show the loss of infectivity of FCoV with a 3 g/L sheep blood/BSA organic load at 20 °C (left) and 4 °C (right). The concentration of infectious FCoV (TCID_50_/germ carrier) is plotted on a logarithmic scale. The symbols represent the actual measurements of infectious FCoV at each time point. At 4 °C with yeast/BSA we underestimated the stability of FCoV and had to start a second series of trials (triangles). Each time point was investigated in triplicate. The red and blue lines indicate posterior draws (uncertainty) of the predicted decay of FCoV from the Bayesian loglog linear regression model for each temperature and organic load combination.

The stability of FCoV when dried in organic matrices on surfaces was assessed using stainless-steel germ carriers with four different combinations of organic loads and temperatures. Its loss of infectivity over time at 4 °C and 20 °C with either a 10 g/L yeast extract and BSA (yeast extract/BSA) load or a 3 g/L sheep blood and BSA (sheep blood/BSA) load is shown in Fig. 3. The days until a reduction of the FCoV concentration by a certain log_10_ level has been achieved are presented in Table 2. The storage location had an average RH of 50% at 4 °C and 18% at 20 °C. On day 1, when yeast extract/BSA or sheep blood/BSA was used as the organic load, we initially measured an FCoV concentration of M = 6.23 or M = 6.43 log_10_ TCID_50_/germ carrier after drying, respectively. The trials were terminated when the concentration of infectious FCoV reached the detection limit of the assay of 1.1 log_10_ TCID_50_/germ carrier. Generally, the loss of infectivity was initially higher and gradually slowed over time. FCoV was most unstable with the yeast extract/BSA load at 20 °C, with a 5 log_10_ TCID_50_ reduction achieved after Md = 19 days (95% CI [16, 21]). In contrast, with the sheep blood/BSA load at 20 °C, a 5 log_10_ TCID_50_ reduction would be reached after Md = 58 days (95% CI [49, 59]). A similar decrease in FCoV concentration was observed with sheep blood/BSA at 4 °C, where a 5 log_10_ TCID_50_ reduction was projected after Md = 54 days (95% CI [47, 75]). Notably, FCoV was most robust with yeast extract/BSA at 4 °C, as the initial concentration decreased significantly slower. It decreased by only 3 log_10_ levels after Md = 68 days (95% CI [57, 79]), remaining infectious three times longer than with sheep blood/BSA at 4 °C. Finally, a 5 log_10_ TCID_50_ reduction was achieved after Md = 167 days (95% CI [155, 180]).

**Table 2:** Days until a reduction of the initial FCoV concentration by a certain log_10_ level has been achieved. FCoV was dried in different organic matrices on stainless-steel surfaces and stored at 20 °C or 4 °C.

Organic load	Storage temperature (°C)	Log_10_ reduction	Median days [95% CI]
Yeast extract/BSA	20	1	2 [2, 3]
Yeast extract/BSA	20	3	7 [6, 8]
Yeast extract/BSA	20	5	19 [16, 21]
Yeast extract/BSA	4	1	8 [6, 10]
Yeast extract/BSA	4	3	68 [57, 79]
Yeast extract/BSA	4	5	167 [155, 180]
Sheep blood/BSA	20	1	3 [3, 3]
Sheep blood/BSA	20	3	13 [11, 15]
Sheep blood/BSA	20	5	58 [47, 75]
Sheep blood/BSA	4	1	4 [3, 5]
Sheep blood/BSA	4	3	21 [18, 25]
Sheep blood/BSA	4	5	54 [49, 59]

*95% CI* 95% confidence interval.

## Discussion

The investigation of virus tenacity in the environment plays a crucial role in enhancing our understanding of potential transmission routes for infectious diseases. Our study focused on assessing the tenacity of airborne FCoV and FCoV in dried organic matrices on surfaces. Overall, airborne FCoV showed a remarkable level of stability over a wide range of RH conditions. However, it is important to note that relative humidity has an impact on FCoV stability. The virus showed higher stability at both low and high RH levels, whereas medium RH conditions (around 50–60%) were associated with a higher probability of decay. Remarkably, FCoV remained infectious for over 7 h in the airborne state at medium RH levels. Moreover, on surfaces, FCoV showed the ability to remain infectious for extended periods, even up to several months. The stability on surfaces was influenced by factors such as temperature and the presence of organic material.

FCoV was used as a surrogate for SARS-CoV-2 in our study. Working with infectious pathogens of biosafety level 3 (SARS-CoV-2) is only possible in a limited number of laboratories, and especially with virus aerosols, it is very challenging. FCoV, on the other hand, can be studied under biosafety level 2 (BSL2) conditions, making it a safer and more cost-efficient option. FCoV belongs to the genus Alphacoronavirus, while SARS-CoV-2 is a Betacoronavirus. Although they share only 44.0–44.5% similarity at their nucleotide level^3^, previous research has shown that non-zoonotic animal coronaviruses like FCoV, canine coronavirus (CCV), transmissible gastroenteritis virus (TGEV) or mouse hepatitis virus (MHV) could be a suitable surrogate for survival of zoonotic SARS-CoVs^27–29^. To the best of our knowledge, this is the first study investigating the tenacity of FCoV in aerosols and on surfaces.

Our aerosol experiments were conducted in an aerosol chamber with a volume of 7 m^3^, allowing individual airflows and climatic conditions. The chamber has been previously utilized in a study investigating the stability of Escherichia coli in aerosols^30^. Other studies investigating pathogen stability in aerosols, including SARS-CoV-2, have utilized a rotating drum, as described by Goldberg et al.^14,31^, to generate a dynamic aerosol. Our chamber offers a good opportunity to create a more realistic setting for exploring pathogen behavior within a room. To investigate FCoV stability, we worked with both dynamic (air exchange) and static (no air exchange) aerosol setups.

We observed a slight U-shaped trend in the stability of FCoV in dynamic aerosols at different RH levels, indicating that FCoV was more likely to decay at medium RH levels, ranging from 50 to 60%. This U-shaped pattern has also been observed for other enveloped viruses, including TGEV and influenza virus^32,33^. However, studies on human coronaviruses have shown varying results. For example, Sars-CoV and MERS have been found to be more stable at medium humidity levels^34,35^. Oswin et al. demonstrated that at low humidity levels, the initial stability decreases significantly but then remains relatively stable compared to higher humidity levels. If this initial decrease is neglected, a U-shaped pattern could also be observed^21^. Overall, coronaviruses in the aerosol state appear to be more stable than influenza or filoviruses at medium humidity levels^34^. When comparing studies on stability of viruses at different humidity levels, it is important to consider the medium used, as significant differences in stability can arise due to this factor. The most important fact to take into consideration when talking about the relationship between stability of viruses and RH is the microenvironment of the droplet and therefore the medium in which it resides^36^. Simulating human respiratory fluids accurately is still challenging due to the unknown exact components and concentrations. Therefore, many studies used cell culture medium such as DMEM as a model medium. One previous study compared DMEM with porcine respiratory fluid (PRF) and found that they differed greatly in the NA:K ratio. In addition, PRF contained significantly more protein^37^. It is important to note that studies using simulated respiratory fluids or real respiratory fluids instead of model medium have shown differences in virus stability^38^. These studies suggest that virus stability might be underestimated in most cases^34,36,39,40^. To make studies more representative, changes should be made to the virus suspension medium in further aerovirology studies^37^.

In our study, we modified DMEM by supplementing it with 10% FBS as a protein source, as respiratory droplets contain a variety of salts and proteins^41,42^. Yang et al. investigated the influence of different model media on the stability of Influenza A viruses in droplets, comparing DMEM and PBS, each with or without the addition of 5% FBS as a protein source. In general, they found better viability in DMEM than in PBS especially at medium and low RH^36^. Notably, the addition of FBS significantly affected virus stability at medium RH levels, suggesting a protective effect of proteins^43,44^. When a droplet leaves the respiratory tract, it evaporates by approximately half its original size depending on the ambient RH. This leads to a high concentration of substances within the droplet, such as salts, which are usually harmless but can become toxic to the virus. This effect is only relevant at medium RH levels just before the salts crystallize^36^. The exact RH at which the salts crystallize (efflorescence RH) depends on the droplet's composition and medium^45^. These findings support our own observations, as we observed a slight decrease in stability at medium RHs in the aerosol. Overall, we observed a high stability of FCoV in the aerosol, likely due to the presence of 10% FBS. The dynamic aerosol setup aimed to simulate a ventilated room where a virus emitter is present. The results indicate that the ambient RH in a room can significantly impact the stability of the emitted virus in the aerosol and thus its transmission potential. To minimize the risk of infection, it is advisable to keep the relative humidity at medium levels in indoor places.

Furthermore, our study demonstrated that FCoV remained infectious in static aerosols for over 7 h with a half-life of 34.8 min. The static aerosol setup aimed to simulate an enclosed room without regular air exchange, where a virus was released for a specific duration. During these experiments, we considered the possible natural loss of virus due to sedimentation. We observed a 31.4% loss of infectious virus through sedimentation, which occurred within the initial 10 min and was than constant over the subsequent 7 h. Moreover, the particle count remained stable throughout the entire experiment, indicating that virus-containing particles relevant for aerosol transmission remained suspended in the aerosol. It is known that aerosol particles < 5 µm, which are relevant for inhalation, remain suspended as droplet nuclei in the air for hours, while larger droplets > 10 µm settle to the ground within minutes due to gravity^11,12,46^. However, re-aerosolization of these sedimented infectious virus particles may also occur. In these experiments, the RH averaged 33%. Previous studies on related viruses have found that SARS-CoV-1 and SARS-CoV-2 remain stable in aerosols for over 3 h, with respective half-lives of 1.1 and 1.2 h, at an RH of 65%^14^. Similarly, MERS-CoV was found to be infectious in aerosols for over 3 h^35^. In our study, we observed that FCoV has a half-life of 34.8 min in aerosols and was detectable for over 7 h. There was one other research group that investigated the stability of SARS-CoV-2 in aerosols over a longer period and found infectious virus after 16 h at an average RH of 53%. However, this was a single observation without replication^18^. Comparing the half-lives of SARS-CoV-2 and FCoV indicates that both exhibit relatively short durations, suggesting similar behavior in aerosol stability. Observed differences may be more likely attributed to different aerosol generation processes and sampling methods. When considering influenza A viruses, their infectivity in aerosols varies lasting from 1 to 24 h, depending on RH levels. Furthermore, influenza A viruses adapted to animals tend to demonstrate longer stability compared to human influenza A viruses^47,48^. It is important to note that comparisons between these studies are challenging due to variations in RH levels and medium used, as both factors strongly influence the stability of airborne viruses, as mentioned earlier. In general, our findings underscore the potential risk of aerosol transmission of enveloped respiratory viruses, especially in enclosed and unventilated environments over an extended period. This aligns with previous studies that have demonstrated aerosol transmission of SARS-CoV-2 between animals using hamsters as an animal model^49,50^.

At optimal environmental conditions the recovery rate of airborne FCoV was approximately 13% in our study. Several factors may have an influence on recovery rates of airborne viruses, including inactivation during aerosolization, loss through sedimentation, as well as sampling losses. We assume that our ultrasonic nebulizer and the aerosilization settings used resulted in the production of a suitable viral aerosol. In a study by Kim et al. various nebulizers and settings like pressure and nebulization time were tested to evaluate their impact on the stability of TGEV, and it was concluded that the stability of TGEV was not significantly affected^32^. Döhla et al. emphasized the importance of selecting an appropriate sampling method, as it can influence the stability of viruses in the sample. Since there is no generally recommended virus air sampling method, the choice of air sampler needs to be individually determined based on the specific experimental setup^51^. Most commonly used air samplers for collecting SARS-CoV-2 include filters, impactors, cyclone samplers and impingers^52^. For our experiments we chose the Coriolis µ cyclone air sampler. Previous studies aiming to detect SARS-CoV-2 in hospitals or healthcare settings have also utilized cyclone samplers due to their high collection volume^53–56^. While SARS-CoV-2 RNA has been detected in these studies, the identification of infectious SARS-CoV-2 was reported in only a few cases. It should be noted that cyclone samplers may be less efficient in detecting low levels of viruses compared to other air samplers, as the centrifugal forces affecting the viruses during collection could potentially cause stress^57^. However, in our study, we worked with high concentrations of viruses in a controlled environment, which made the Coriolis µ sampler suitable for our purposes, and we were able to detect infectious viruses.

We observed that 31.4% of the infectious virus sedimented onto the ground or surfaces within the first 10 min in the static aerosol. This finding highlights the potential risk of contact transmission and the importance of studying virus infectivity on commonly encountered surfaces. We focused on stainless steel surfaces, which are frequently found in public buildings and clinical settings and are frequently touched. Previous studies have shown that CoVs exhibit greater stability on non-porous surfaces like metal, glass or plastic compared to porous surfaces, such as paper or fabrics^58,59^. Furthermore, viruses tend to be more stable at lower humidity levels and temperatures^59^. In our study, we demonstrated that FCoV remained infectious for 19–58 days at 20 °C and low RH, with the organic load significantly influencing the virus's stability. Comparatively, SARS-CoV-2 remained infectious on stainless steel surfaces for 4–7 days at room temperature, while MERS and Sars-CoV-1 remained infectious for 2 days^14,15,35,60,61^. TGEV and MHV, other non-zoonotic CoVs, remained infectious at room temperature for 3 days at 50% RH and up to 28 days at 20% RH^27^. It is important to note that differences in the results of various studies may occur due to varying medium used. While most of these studies were conducted using cell culture medium, we enriched our medium with 10 g/L yeast extract/BSA or 3 g/L sheep blood/BSA, representing a high organic load according to the guidelines for virus inactivation studies on nonporous surfaces^62^. Exhaled droplets that would sediment on surfaces consist of respiratory tract residues, saliva and organic material from the environment, resulting in a high organic load. Other studies added a tripartite soil load (mucin, BSA and tryptone) following international standard ASTM to the medium and found increased stability of SARS-CoV-2 on stain-less steel surfaces at 20 °C for 14–28 days, indicating a protective effect of the organic load^16,63^. Therefore, we would suggest using a high organic load, such as ASTM International’s standardized tripartite soil load^64^, for further studies to avoid underestimating the stability of these viruses in the environment. However, it should be taken into consideration that stability may differ in dried human respiratory fluids. Regarding the influence of temperature, we found that infectious FCoV was detectable at 4 °C and 50% RH for 54–167 days, depending on the organic load. Only few studies have investigated CoVs stability at temperatures below 20°. Notably, Onianwa et al. observed a reduction in infectiousness of the Delta variant of SARS-CoV-2 at 24 °C and 65% RH in the first 2.5 h, while no reduction was observed at 4 °C and 85% RH within 2.5 h^65^. TGEV and MHV also remained infectious at 4 °C for over 28 days at all tested RHs, with the lowest losses observed at 20% RH^27^. Interestingly, we observed prolonged infectivity with yeast extract at 4 °C, although the reason for this difference remains unclear.

Like in aerosols, evaporation, and thus RH, plays an important role in terms of virus stability in droplets that sediment. French et al. studied the interplay of droplet volume and RH on surfaces and found that loss of infectivity was slower and more affected by RH in larger droplets (50 µL) than in small droplets (1 µL)^66^. Studies investigating stability of CoVs on surfaces, including our study, all used larger droplet volumes, which are not in line with realistically expelled droplet volumes (< 0.5 µL) and may lead to different conclusions about virus stability. Another limitation of our study design is that we could not regulate the RH at the storage place and therefore could not distinguish between the influence of temperature and RH after drying. However, French et al. found that that viral decay during the wet phase was higher than during the dry phase regardless of RH^66^. In our experiment, all germ carriers were dried under controlled conditions for 45 min, allowing us to neglect the influence of RH during the wet phase. Thus, the observed differences in stability may be primarily attributed to temperature and variations in organic load.

In summary, our study demonstrated that FCoV could remain infectious in the airborne state for hours and on surfaces up to months, with the duration depending on environmental conditions. Factors such as RH, temperature, and the presence of organic material significantly impact the pathogen's infectivity outside the host. Comparing studies on virus stability is challenging due to the lack of standardized experimental setups and medium used in these investigations. Additionally, reproducing respiratory fluids in the laboratory is difficult as their exact composition is still unknown. However, existing evidence suggests that viruses may exhibit even greater stability in respiratory fluids. It can be stated that aerosol transmission as well as droplet and contact transmission are possible transmission routes for coronaviruses under various environmental conditions over an extended period. Whether an infection occurs depends on many other factors, such as the viral load in the environment, the minimum infection dose, and the immune state of individuals. Especially enclosed, poorly ventilated rooms and low RH environments may pose a higher risk of infection due to the accumulation and better stability of these enveloped viruses. Given that, different pathogens respond uniquely to environmental conditions based on their biological and physical properties, it is essential to study a wide range of viruses to identify and understand potential correlations. The exact mechanisms that lead to the inactivation or protection of enveloped viruses by environmental components remain unknown and require further research. Our study suggests that FCoV could be a valuable surrogate for studying the behavior of zoonotic coronaviruses like SARS-CoV-2 in the environment. Although surrogates could offer valuable insights into the stability and persistence of these viruses outside the host, enhancing our understanding of zoonotic transmission dynamics, it remains crucial to directly investigate the actual virus.

## Data Availability

The data that support the findings of this study are available in the figures and tables of this paper. Raw data is available on request from the authors.
